# Supplementary material for: Prolonged T-cell activation and long COVID symptoms independently associate with severe COVID-19 at 3 months
Source: eLife. 2023 Jun 13;12:e85009. doi: 10.7554/eLife.85009 (PMC10319436; doi:10.7554/eLife.85009)
Supplement: Figure 6—source data 1. — p-Values are shown with or without FDR correction (fdr_p or p values, respectively) for both adjusted and unadjusted models. Highlighted in grey are the two immune parameters that significantly correlated with symptoms after FDR correction. Only parameters with FDR uncorrected p-values <0.05 are included in the tables. [file elife-85009-fig6-data1.docx]

**Figure 6- source data 1**

**A**

| Immune parameter​ | Outcome​ | Estimate​ | Standard error ​ | p.value​ | Model​ | fdr_p​ |
| --- | --- | --- | --- | --- | --- | --- |
| CD4+ T cell cluster 2 | Symptoms | 0.04576367​ | 0.01119604​ | 4.36E-05​ | 12 week​ | 0.0031397​ |
| CD8+ T cell cluster 4 ​ | Symptoms | 0.54878833​ | 0.15569149​ | 4.24E-04​ | 12 week​ | 0.01525431​ |
| % CD4+ TCM Ki67+ HLA DR+ cells​ | Symptoms | -0.3065684​ | 0.11515405​ | 0.00776212​ | 12 week​ | 0.17564759​ |
| Ratio CD4+/CD8+ T cells​ | Symptoms | 0.13596711​ | 0.05261311​ | 0.0097582​ | 12 week​ | 0.17564759​ |
| CD4+ T cell cluster 11​ | Symptoms | -0.4074559​ | 0.17224934​ | 0.01800571​ | 12 week​ | 0.25899435​ |
| % CD8+ naive granz+ CD38​+ | Symptoms | -0.0513135​ | 0.02233379​ | 0.02158286​ | 12 week​ | 0.25899435​ |
| % CD8+ naive HLA-DR+ CD38+ | Symptoms | -0.092843​ | 0.04410418​ | 0.03528394​ | 12 week​ | 0.32171462​ |
| % CD4+ TCM HLA-DR+ CD38+​ | Symptoms | -0.1199945​ | 0.0572781​ | 0.03617586​ | 12 week​ | 0.32171462​ |
| % CD4+ naive Ki67+ HLA-DR+ ​ | Symptoms | -0.3038127​ | 0.14981782​ | 0.0425724​ | 12 week​ | 0.32171462​ |
| % CD4+ Ki67+ CD38+ | Symptoms | -0.256065​ | 0.1296416​ | 0.04824815​ | 12 week​ | 0.32171462​ |

**B**

| Immune parameter​ | Outcome​ | Estimate​ | Standard error | p.value​ | Model​ | fdr_p​ |
| --- | --- | --- | --- | --- | --- | --- |
| IP-10 | Symptoms | 0.03252012​ | 0.01099191​ | 0.00309088​ | 12 week​ | 0.15074485​ |
| CD4+ T cell cluster 8 | Symptoms | 1.40814142​ | 0.50904306​ | 0.00567047​ | 12 week​ | 0.15074485​ |
| % CD4+ TEM Ki67+ HLA-DR+ | Symptoms | 0.11740078​ | 0.04296087​ | 0.00628104​ | 12 week​ | 0.15074485​ |
| % CD4+ TCM granz+ CD38​+ | Symptoms | -8.4815195​ | 3.36641524​ | 0.01175379​ | 12 week​ | 0.21156817​ |
| Il-4 | Symptoms | 0.01318671​ | 0.00577716​ | 0.0224563​ | 12 week​ | 0.28171972​ |
| IFN-g | Symptoms | 0.00959219​ | 0.00423471​ | 0.02350424​ | 12 week​ | 0.28171972​ |
| Il-12 | Symptoms | 0.00386354​ | 0.00175144​ | 0.02738942​ | 12 week​ | 0.28171972​ |
| CD8+ T cell cluster 3 | PCS | -0.14212855 | 0.06285575 | 0.03091156 | 12 week​ | 0.92070476 |
| CD8+ T cell cluster 3 | MCS | -0.14212855 | 0.06285575 | 0.03091156 | 12 week​ | 0.92070476 |
| % CD4+ TCM HLA-DR+ CD38+ | Symptoms | -0.1999689​ | 0.09984217​ | 0.04519338​ | 12 week​ | 0.92070476 |
